# A SelectMDx/magnetic resonance imaging‐based nomogram to diagnose prostate cancer

**DOI:** 10.1002/cnr2.1668

**Published:** 2022-09-27

**Authors:** Vinayak G. Wagaskar, Micah Levy, Parita Ratnani, Sharmila Sullimada, Mae Gerenia, Kacie Schlussel, Samia Choudhury, Marla Gabriele, Ian Haas, Kenneth Haines, Ash Tewari

**Affiliations:** ^1^ Department of Urology Icahn School of Medicine at Mount Sinai Hospital New York New York USA; ^2^ Department of Pathology Icahn School of Medicine at Mount Sinai Hospital New York New York USA

**Keywords:** mpMRI PI RADS score, PCa biomarkers, prostate biopsy, SelectMDx

## Abstract

**Background:**

70%–80% of prostate cancer (PCa) biopsies performed in the US annually may be unnecessary. Specific antigen testing (PSA) and tans rectal ultrasound (TRUS) are imprecise predictive methods for risk of PCa. Novel strategies are critical to guide biopsy decision‐making.

**Aim:**

We assessed the utility and accuracy of combining Select MDx and multiparametric magnetic resonance imaging (mpMRI) scores for predicting risk of PCa.

**Methods and Results:**

Our study was conducted at Mount Sinai hospital at Urology department in New York City from January 2020 to April 2021. Total 129 men performed select MDx test. Indications for prostate biopsy were high‐risk Select MDx score, suspicious DRE, PI‐RADS scores 3/4/5 on mpMRI, or any combination of these. Fifty‐one percentage of 129 patients underwent systemic or combined systemic and MRI/US (ultrasound) fusion biopsy; All men underwent 3 T MRI of Prostate w/wo contrast using standard protocols prior to biopsy. A single surgeon performed prostate biopsies.

Gleason score ≥3 + 3 on biopsy is defined as outcome. Descriptive statistics were calculated as cross tables. Binary logistic regression model is used to determine the outcome. The nomogram was based on the coefficients of the logit function. ROCs were plotted and decision curve analysis was performed.

Using both high‐risk Select MDx and PI‐RADS scores of 4/5, 87% of biopsies could have been avoided, while detecting 64% of PCa and missing 36%. If biopsies were performed on men with positive Select MDx or PI‐RADS 4/5 results, 16% of biopsies could have been avoided while detecting all PCa. Combining these scores improved specificity and accuracy for the detection of PCa over either used alone. Study limitations include limited sample size, sole institution study, and risk or overfitting for the proposed model which may limit generalizability.

**Conclusion:**

Combining SelectMDx and mpMRI PI‐PADS scores of 4/5 may be useful for PCa biopsy decision‐making.

## 1 BACKGROUND

The poor diagnostic performance of prostate specific antigen (PSA) testing and the cancer agnostic nature of conventional Trans Rectal Ultrasound (TRUS) guided biopsies have led to a long‐standing debate about prostate cancer (PCa) diagnosis.^1^, ^2^ Roughly 1–2 million prostate biopsies are carried out in the United States every year, creating a tremendous strain on the health care system. Yet, it has been estimated that as many as 70–80% of biopsies may be unnecessary.^3^, ^4^, ^5^ No less significantly, transrectal biopsies are associated with infectious complications in 7%–8% of patients, requiring hospitalization in approximately 3% of patients.^6^ Advances in magnetic resonance imaging (mpMRI) has helped in guiding the decision of the prostate biopsy and biopsy techniques.^7^ However, MRI still misses clinically significant prostate cancer (Gleason score ≥3 + 4) in 4%–18% men.^8^, ^9^, ^10^ A number of blood, body fluid, and tissue‐based biomarkers have been developed with the aim of estimating the likelihood of disease characteristics, more accurately determining prognosis, or predicting response to a specific treatment.^11^

The Select MDx assay (MDx Health) combines expression levels from HOXC6 (a prostate cancer progression gene), DLX1 (a prostate cancer cell proliferation gene), KLK3 (a prostate cancer reference gene) along with clinical parameters such as age, digital rectal examination [DRE] findings, PSA levels and prostate volume, in a logistic regression model.^12^ Select MDx is a reverse‐transcription polymerase chain reaction (RT‐PCR) assay performed on urine specimens collected immediately following digital examination (DRE) from patients being considered for prostate biopsy. The current study focuses on the utility of the Select MDx score combined with the mpMRI score for diagnosing the risk of prostate cancer (PCa).

## 2 METHODS

Our study was conducted at Mount Sinai hospital at Urology department after an IRB approval. In this retrospective study, we evaluated patients records that underwent biopsy between January 2020 and April 2021. We found 129 patients had performed Select MDx test. Of these patients, 51 (40%) underwent systematic or combined systematic and MRI/ultrasound (US) fusion biopsy. Candidates were selected for biopsy if he has abnormal rectal examination of prostate, high‐risk Select MDx score or PI‐RADS scores of 3, 4 or 5 on mpMRI, or any combination of these measures. 3 T MRI of Prostate w/wo contrast using standard protocols prior to biopsy. MRI results were evaluated according to PI‐RADS Version 2 (PI‐RADS V2) by radiologists with minimum 5 years of experience.^13^

Spring‐driven 18‐gauge caliber biopsy gun is used for performing biopsies. Artemis MRI/TRUS fusion device (Innomedicus, Cham, Switzerland) was used for targeted biopsies. All biopsies were performed by senior author [A.K.T.] and were reviewed by a pathologist (K.H. III).

### 2.1 Outcome definition and statistical analysis

Gleason score ≥3 + 3 on biopsy is defined as outcome and were considered as cases. Men with no cancer on biopsy considered controls. Descriptive statistics were calculated as cross tables. The prediction model includes the Select MDx and mpMRI PI‐RADS scores. Because the Select MDx score incorporates age, DRE, PSA, and prostate volume, we did not include these variables in building the model. Binary logistic regression model is used to determine the outcome. A nomogram was then built based on the coefficients of the logit function. The receiver‐operating characteristics (ROCs) were plotted using same variables. Decision curve analysis (DCA) is performed to evaluate model performance. SPSS v.27.0 (IBM Corp., Armonk, NY, USA) and STATA 12 (StataCorp LP, College Station, TX, USA) were used in analysis. *p* < .05 considered as statistically significant.

## 3 RESULTS

Of 51 men, 14 (28%) had cancer on biopsy and 37 (72%) did not show evidence of cancer. Median age was 63 years (interquartile range [IQR] 60, 66) and 71 years (IQR 67, 75) for benign biopsies and PCa, respectively; median PSA was 5.8 ng/ml (IQR 4.5, 6.4), 6.4 ng/ml (IQR 5.4, 7.7); and median PSA density was 0.07 (IQR 0.06, 0.08), 0.09 (IQR 0.08, 0.10) for men with benign biopsy and men with PCa, respectively. The Select MDx score was high risk in 16 (43%) and 13 (93%) of men with benign biopsies and men with PCa on biopsies, respectively. Of the 14 men with PCa, 7 (50%), 4 (29%), 2 (14%), and 1 (7%) had ISUP Gleason grades of 1, 2, 3 and 5, respectively.

### 3.1 Univariable and multivariable analysis predicting PCa

On univariate analysis, age and the Select MDx score were found to be significant predictors of PCa (Table 1). On multivariate analysis, Select MDx and MRI PI‐RADS scores of 4 and 5 were significantly associated with PCa (Table 2).

**TABLE 1:** Comparison of factors between cases and controls

Variable	Benign biopsy, *n* = 37	Cancer on biopsy, *n* = 14	*p* Value
Median Age in years	63 (60,66)	71 (67, 75)	0.032^a^
Median BMI in kg/m^2^	27 (26,28)	28 (27, 29)	0.310
Race			0.067
Caucasian	34 (92)	13 (93)	
AA	‐	1 (7)	
Others	3 (8)	‐	
Family history of PCa			0.416
No	28 (76)	9 (64)	
Yes	9 (24)	5 (36)	
DRE			0.988
Normal	29 (78)	11 (79)	
Suspicious	8 (22)	3 (21)	
Median PSA in ng/dl	5.8 (4.5, 6.4)	6.4 (5.4, 7.7)	0.687
Median PSA density	0.07 (0.06,0.08)	0.09 (0.08,0.10)	0.087
Select MDx			0.001^a^
Very low risk	21 (57)	1 (7)	
High risk	16 (43)	13(93)	
MRI Prostate volume cc	64 (58, 68)	67 (59, 71)	0.969
MRI PI‐RADS score			0.062
0	12 (32)	2 (14)	
3	14 (38)	2 (14)	
4–5	11 (30)	10(72)	
ISUP grade group			0.000^a^
0	37 (100)	0	
1	0	7 (50)	
2	0	4 (29)	
3	0	2 (14)	
4	0	0	
5	0	1 (7)	

Abbreviations: AA, African Americans; BMI, body mass index; DRE, digital rectal examination findings; ISUP, International Society of Urologic Pathology; MRI, magnetic resonance imaging; PCa, prostate cancer; PI‐RADS score, Prostate Imaging and Reporting Data System v.2 score; PSA, prostate specific antigen.

^a^ *p* value <.05.

**TABLE 2:** Multivariable logistic regression analysis predicting prostate cancer

Variable	Estimates	Odds ratio	95% CI for odds ratio		*p* Value
			Lower	Upper	
Select MDx score	2.85	17.31	1.89	158.11	0.01^a^
MRI PI‐RADS score					
PI‐RADS 3	0.13	1.12	0.12	10.63	0.91
PI‐RADS 4/5	1.84	6.35	1.11	14.12	0.04^a^

Abbreviations: CI, confidence intervals; MRI, magnetic resonance imaging; PI‐RADS score, Prostate Imaging and Reporting Data System v.2 score.

^a^ *p* Value <.05.

### 3.2 Nomogram to estimate the risk of PCa

**FIGURE 1:**
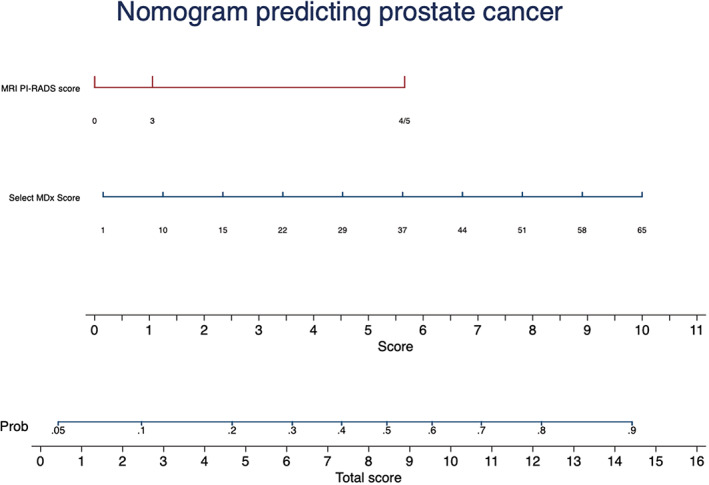
Nomogram prediction model for predicting prostate cancer after prostate biopsy. The prediction of adverse pathology probability from the nomogram includes the following steps: (1) Locate the patient's variable MRI PI‐RADS score on the corresponding axis. (2) Draw a line straight down to the score axis to determine how many points toward the probability of cancer the patient receives for his PIRADS score. (3) Repeat the process for the Select MDx score. (4) Total the points for each of the predictors. (5) Locate the final sum on the total score axis. (6) Draw a line straight up to find the patient's probability [Prob] of having Prostate cancer. MRI PIRADS, magnetic resonance imaging Prostate Imaging and Reporting Data System v.2 score; PI‐RADS 0, No PI‐RADS score or PI‐RADS score 1 or 2.

**FIGURE 2:**
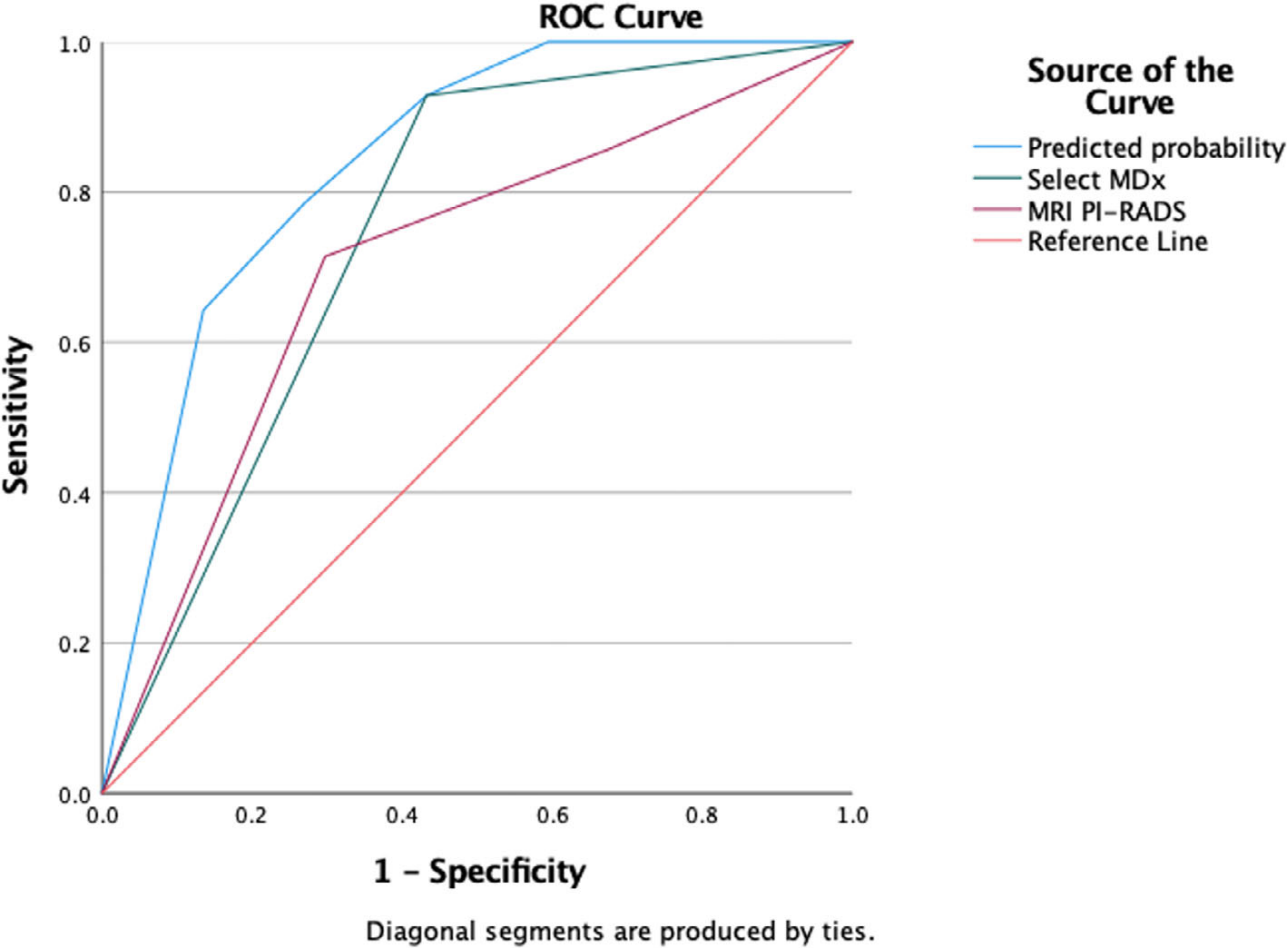
Area under curve characteristics of the prediction model for prostate cancer. PIRADS, magnetic resonance imaging Prostate Imaging and Reporting Data System v.2 score.

**FIGURE 3:**
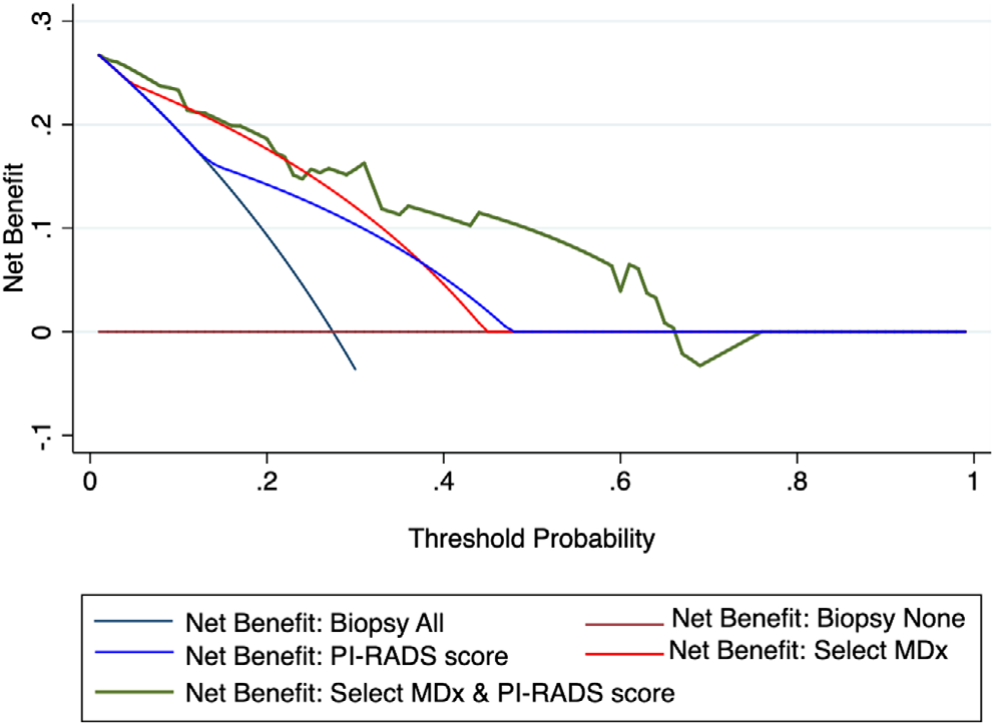
Decision curve analyses showing the net benefit associated with the use of nomogram‐derived probability for prediction of prostate cancer rather than relying on Select MDx score or PI‐RADS score information only. PIRADS, magnetic resonance imaging Prostate Imaging and Reporting Data System v.2 score.

Figure 1 shows utility of the Select MDx score and mpMRI PI‐RADS scores 3/4/5 in building nomogram for prediction of PCa. Higher Select MDx score and PI‐RADS scores of 4/5 has increased probability of finding cancer on prediction of PCa. Area under curve (AUC) for predicting PCa was 0.84 which was higher than Select MDx score or MRI PI‐RADS score alone (Figure 2). Both Select MDx and MRI PI‐RADS score has contributed to building the AUC of the model. Decision curve analysis (DCA) demonstrated better clinical risk prediction using the Select MDx and mpMRI PI‐RADS scores for predicting prostate cancer vs relying on the MRI PI‐RADS score or Select MDx alone (Figure 3).

### 3.3 Sensitivity, specificity, positive predictive value, negative predictive value, and accuracy of the analysis

We evaluated the performance of utilizing Select MDx and mpMRI PI‐RADS scores of 4/5 for predicting PCa at the time of biopsy (Table 3A). Sensitivity (Sn), specificity (Sp), positive predictive value (PPV), negative predictive value (NPV), and the accuracy of the Select MDx only was 93%, 57%, 45%, 95% and 67%, respectively for predicting PCa at the time of biopsy. Sn, Sp, PPV, NPV and the accuracy of an MRI PI‐RADS score of 4/5 was 71%, 70%, 48%, 87%, and 71%, respectively for predicting PCa at the time of biopsy. Combining Select MDx and PI‐RADS 4/5 scores resulted in Sn, Sp, PPV, and NPV accuracy of 64%, 87%, 64%, 87% and 80%, respectively for predicting PCa at the time of biopsy while, utilizing either Select MDx or PI‐RADS 4/5 scores resulted in Sn, Sp, PPV, and NPV accuracy of 100%, 16%, 28%, 100% and 39%, respectively for predicting PCa at the time of biopsy. In non‐suspicious MRI findings (i.e., no PI‐RADS score or PI‐RADS score of 1, 2), Sn, Sp, PPV, NPV, and accuracy of the Select MDx score was 14%, 84%, 25%,72%, and 65% respectively for predicting PCa at the time of biopsy. A very low‐risk Select MDx score, Sn, Sp, PPV, NPV, and accuracy of an MRI PI‐RADS score of 4/5 was 3%, 68%, 14%, 30%, and 28%, respectively for predicting PCa at the time of biopsy.

**TABLE 3:** Performance of select MDx and PI‐RADS score in different clinical scenarios

(A) Variable included for biopsy	Sn	Sp	PPV	NPV	Accuracy
Select MDx only	93	57	45	95	67
PI‐RADS 4/5 only	71	70	48	87	71
Select MDx or PI‐RADS 4/5	100	16	28	100	39
Select MDx and PI‐RADS 4/5	64	87	64	87	80
Select MDx + non‐suspicious MRI	14	84	25	72	65
PI‐RADS 4/5 + very low risk Select MDx	3	68	14	30	28

(B) Biopsy selection variable	Avoided biopsy, *n* = 37 (%)	PCa, *n* = 14(%)	
		Detected (%)	Missed (%)
Select MDx only	21 (57)	13 (93)	1 (7)
PI‐RADS 4/5 only	26 (70)	10 (71)	4 (29)
Select MDx or PI‐RADS 4/5	6 (16)	14 (100%)	0 (0)
Select MDx and PI‐RADS 4/5	32 (87)	9 (64)	5 (36)
Select MDx + non‐suspicious MRI	26 (70)	2 (14)	12 (86)
PI‐RADS 4/5 + very low risk Select MDx	31 (84)	1 (7)	13(93)

Abbreviations: MRI, magnetic resonance imaging; NPV, negative predictive value; PI‐RADS, Prostate Imaging and Reporting Data System v.2 score; PPV, positive predictive value; Sn, sensitivity; Sp, specificity.

Employing different strategies utilizing Select MDx and mpMRI PI‐RADS scores we calculated the number of biopsies that could be avoided, missed PCa, and detected PCa (Table 3B). With high‐risk Select MDx alone, 57% of biopsies could have been avoided, while missing 7% of PCa and detecting 93% of PCa. Using a PI‐RADS score of 4/5 alone at the time of biopsy could have avoided 70% biopsies, while missing 29% PCa and detecting 71% of PCa. Using both a high‐risk Select MDx score and a PI‐RADS score of 4/5, 87% of biopsies could have been avoided while detecting 64% PCa and missing only 36% PCa. Using either a high‐risk Select MDx result or a PI‐RADS score of 4/5, 16% of biopsies could have been avoided while detecting 100% of PCa (14/14). Using a strategy assessing non‐suspicious mpMRI and high‐risk Select MDx scores could have avoided 70% of biopsies but would have missed 86% of PCa, detecting only 14% of PCa. Utilizing a very low‐risk Select MDx score and a mpMRI PI‐RADS score of 4/5 could have avoided 84% of biopsies while missing 93% of PCa and detecting only 7% PCa.

## 4 DISCUSSION

Substantial efforts continue to find methods to improve PCa diagnosis. We have developed a prognostic tool for the primary diagnosis of prostate cancer at the time of biopsy combining Select MDx and mpMRI PI‐RADS scores. Our Select MDx/mpMRI PI‐RADS model confers three key benefits: (1) reduction in the number of unnecessary biopsies; (2) efficacy for predicting PCa; (3) improved specificity (Sp) and accuracy in detecting PCa vs relying on the PI‐RADS or Select MDx scores alone.

Our findings show that inclusion of pre‐biopsy level genomic information in risk stratification increases the accuracy of prostate cancer diagnostics. This finding will serve as a platform for future studies to explore the integration of genetic information into the creation of more accurate risk prediction models in prostate cancer, hopefully leading to improved treatment allocation.

Numerous PCa biomarkers have been developed and commercialized either alone or in combination with clinical tools for more effective detection of PCa. The Select MDx score used for prostate cancer detection in this study incorporates genomic markers, along with age, PSA, prostate volume, and DRE findings. Studies have shown that SelectMDx scores can improve detection of PCa with AUC ranging from 0.82 to 0.85.^12^, ^14^ Our study confirms the significant role of the Select MDx score in detecting PCa with AUC 0.74 (alone) or 0.84 (combination with mpMRI). Additionally, multiple studies have shown a role for mpMRI in the detection of PCa rendering it the imaging modality of choice.^7^, ^10^ Studies have shown the significance of PI‐RADS scores of 4 and 5 for the detection of PCa.^15^, ^16^, ^17^, ^18^, ^19^ Our study also confirms these findings.

The Select MDx score when combined with the mpMRI PI‐RADS score have increased percentages of missed cancers; but it has also avoided 87% of unnecessary prostate biopsies. Common non‐fatal complications after biopsy are pain, bleeding, and voiding dysfunction. Post‐biopsy fever and infection are less common, but potentially fatal, complications.^20^ We have also seen a rising prevalence of antibiotic‐resistant bacterial infections with biopsy‐related infectious complications.^21^ Combining select‐MDx score with PI‐RADS 4/5 would help in avoiding these unnecessary biopsies. The Select MDx sensitivity and specificity from this study are consistent with large, multicentre validation studies^22^, ^23^ The negative predictive value (NPV) of mpMRI for diagnosing any cancer in our study was 87%, which is well within published results.^22^ The highest NPV and PPV were obtained with when both Select MDx and mpMRI results were negative or both tests positive (100% and 64%, respectively).

We recognize a number of limitations in our study. Firstly, limited sample size and number of events (14 PCa cases) may affect its applicability. Second, this is a retrospective, one institution experience, hence the outcomes may not be reproducible. Additionally, the limited number of events and the risk of overfitting for the proposed model may affect its generalizability and applicability.

## 5 CONCLUSION

We built a model based on pre‐biopsy level genomic and imaging information to help clinicians to select appropriate candidates for prostate biopsy. This will serve as a platform to integrate genomic information with radiological features for upcoming studies.

## AUTHOR CONTRIBUTIONS

*Data Curation, Formal Analysis, Investigation, Resources*, M.L.; *Data Curation, Methodology, Supervision*, P.R.; *Formal Analysis, Methodology, Resources, Supervision*, S.S.; *Data Curation, Investigation, Visualization*, M.G.; *Formal Analysis, Investigation, Supervision*, K.S.; *Formal Analysis, Methodology, Resources*, S.C.; *Resources, Validation, Visualization*, M.G.; *Formal Analysis, Resources, Visualization*, I.H.; *Data Curation, Validation, Writing—Review and Editing*, K.H.

## CONFLICTS OF INTEREST

Dr. A. K. Tewari has served as a site‐PI on pharma/industry‐sponsored clinical trials from Kite Pharma, Lumicell, Inc., Dendreon, and Oncovir, Inc. He has received research funding (grants) to his institution from DOD, NIH, Axogen, Intuitive Surgical, AMBFF, and other philanthropy. Dr. A. K. Tewari has served as an unpaid consultant to Roivant Biosciences and advisor to Promaxo. He owns equity in Promaxo.

## ETHICS STATEMENT

Our study was conducted at Mount Sinai hospital at Urology department after an IRB [GCO 19‐1711].

## Supporting information


**Supplimentary Figure** Case scenario showing patient information, Select MDx score information, MRI information and biopsy information. 73 years old gentleman with PSA 4.5ng/dL and elevated Select MDx score underwent MRI prostate that showed PI‐RADS 4 on right midgland of prostate. Nomogram showed 45% risk of prostate cancer.Patient underwent biopsy and found to have Gleason 3+4 prostate cancer.

## Data Availability

Corresponding author can provide the data upon reasonable request.

## References

[1] Meigs JB , Barry MJ , Oesterling JE , Jacobsen SJ . Interpreting results of prostate‐specific antigen testing for early detection of prostate cancer. J Gen Intern Med. 1996;11(9):505‐512. 8905498 10.1007/BF02599596

[2] Harvey CJ , Pilcher J , Richenberg J , Patel U , Frauscher F . Applications of transrectal ultrasound in prostate cancer. Br J Radiol. 2012;85(1):S3‐S17. doi:10.1259/bjr/56357549 22844031 PMC3746408

[3] Voigt JD , Zappala SM , Vaughan ED , Wein AJ . The kallikrein panel for prostate cancer screening: its economic impact. Prostate. 2014;74:250‐259. 24166488 10.1002/pros.22746

[4] Loeb S , Carter HB , Berndt SI , Ricker W , Schaeffer EM . Complications after prostate biopsy: data from SEER‐Medicare. J Urol. 2011;186:1830‐1834. 21944136 10.1016/j.juro.2011.06.057 PMC9840843

[5] Halpern JA , Shoag JE , Artis AS , et al. National trends in prostate biopsy and radical prostatectomy volumes following the United States preventive services task force guidelines against prostate‐specific antigen screening. JAMA Surg. 2017;152:192‐198. 27806151 10.1001/jamasurg.2016.3987

[6] Wagenlehner FM , van Oostrum E , Tenke P , et al. Infective complications after prostate biopsy: outcome of the Global Prevalence Study of Infections in Urology (GPIU) 2010 and 2011, a prospective multinational multicenter prostate biopsy study. Eur Urol. 2013;63(3):521‐527. 22704727 10.1016/j.eururo.2012.06.003

[7] Wagaskar VG , Sobotka S , Ratnani P , et al. A 4K score/MRI‐based nomogram for predicting prostate cancer, clinically significant prostate cancer, and unfavorable prostate cancer. Cancer Rep (Hoboken). 2021;4(4):e1357. doi:10.1002/cnr2.1357 33661541 PMC8388161

[8] Bryant RJ , Hobbs CP , Eyre KS , et al. Comparison of prostate biopsy with or without prebiopsy multiparametric magnetic resonance imaging for prostate cancer detection: an observational cohort study. J Urol. 2019;201:510‐519. 30266332 10.1016/j.juro.2018.09.049

[9] Wysock JS , Mendhiratta N , Zattoni F , et al. Predictive value of negative 3T multiparametric magnetic resonance imaging of the prostate on 12‐core biopsy results. BJU Int. 2016;118:515‐520. 26800439 10.1111/bju.13427

[10] Wagaskar VG , Levy M , Ratnani P , et al. Clinical utility of negative multiparametric magnetic resonance imaging in the diagnosis of prostate cancer and clinically significant prostate cancer. Eur Urol Open Sci. 2021;28:9‐16. 34337520 10.1016/j.euros.2021.03.008 PMC8317880

[11] Porzycki P , Ciszkowicz E . Modern biomarkers in prostate cancer diagnosis. Cent European J Urol. 2020;73(3):300‐306. doi:10.5173/ceju.2020.0067R PMC7587476 33133657

[12] Hendriks RJ , van der Leest MMG , Dijkstra S , et al. A urinary biomarker‐based risk score correlates with multiparametric MRI for prostate cancer detection. Prostate. 2017 Oct;77(14):1401‐1407. doi:10.1002/pros.23401 28853167

[13] Weinreb JC , Barentsz JO , Choyke PL , et al. PI‐RADS prostate imaging—reporting and data system: 2015, version 2. Eur Urol. 2016;69:16‐40. 26427566 10.1016/j.eururo.2015.08.052 PMC6467207

[14] Van Neste L , Hendriks RJ , Dijkstra S , et al. Detection of high‐grade prostate cancer using a urinary molecular biomarker‐based risk score. Eur Urol. 2016 Nov;70(5):740‐748. doi:10.1016/j.eururo.2016.04.012 27108162

[15] Cash H , Maxeiner A , Stephan C , et al. The detection of significant prostate cancer is correlated with the prostate imaging reporting and data system (PI‐RADS) in MRI/transrectal ultrasound fusion biopsy. World J Urol. 2016;34(4):525‐532. doi:10.1007/s00345-015-1671-8 26293117

[16] Faiella E , Santucci D , Greco F , et al. Analysis of histological findings obtained combining US/mp‐MRI fusion‐guided biopsies with systematic US biopsies: mp‐MRI role in prostate cancer detection and false negative. Radiol Med. 2018;123(2):143‐152. doi:10.1007/s11547-017-0814-y 29019021

[17] Wagaskar VG , Lantz A , Sobotka S , et al. Development and external validation of a prediction model to identify candidates for prostate biopsy. Urol J. 2022;1. doi:10.22037/uj.v18i.6852 34978065

[18] Jiang S , Huang Z , Liu B , et al. MRI‐based nomogram of prostate maximum sectional area and its zone area for prediction of prostate cancer. Frontiers. Oncology. 2021;11. doi:10.3389/fonc.2021.708730 PMC8458948 34568034

[19] Chau EM , Russell B , Santaolalla A , et al. MRI‐based nomogram for the prediction of prostate cancer diagnosis: a multi‐centre validated patient–physician decision tool. J Clin Urol. 2022. 20514158211065949.

[20] Borghesi M , Ahmed H , Nam R , et al. Complications after systematic, random, and image‐guided prostate biopsy. Eur Urol. 2017;71(3):353‐365. 27543165 10.1016/j.eururo.2016.08.004

[21] Aly M , Dyrdak R , Nordström T , et al. Rapid increase in multidrug‐resistant enteric bacilli blood stream infection after prostate biopsy—a 10‐year population‐based cohort study. Prostate. 2015;75(9):947‐956. 25808608 10.1002/pros.22979

[22] Haese A , Trooskens G , Steyaert S , et al. Multicenter optimization and validation of a 2‐gene mRNA urine test for detection of clinically significant prostate cancer before initial prostate biopsy. J Urol. 2019;202(2):256‐263. doi:10.1097/JU.0000000000000293 31026217

[23] Hendriks RJ , van der Leest MMG , Israël B , et al. Clinical use of the SelectMDx urinary‐biomarker test with or without mpMRI in prostate cancer diagnosis: a prospective, multicenter study in biopsy‐naïve men. Prostate Cancer Prostatic Dis. 2021;24(4):1110‐1119. 33941866 10.1038/s41391-021-00367-8 PMC8616754

