# Supplementary figures and images for: A SelectMDx/magnetic resonance imaging‐based nomogram to diagnose prostate cancer
Source: Cancer Rep (Hoboken). 2022 Sep 27;6(1):e1668. doi: 10.1002/cnr2.1668 (PMC9875685; doi:10.1002/cnr2.1668)

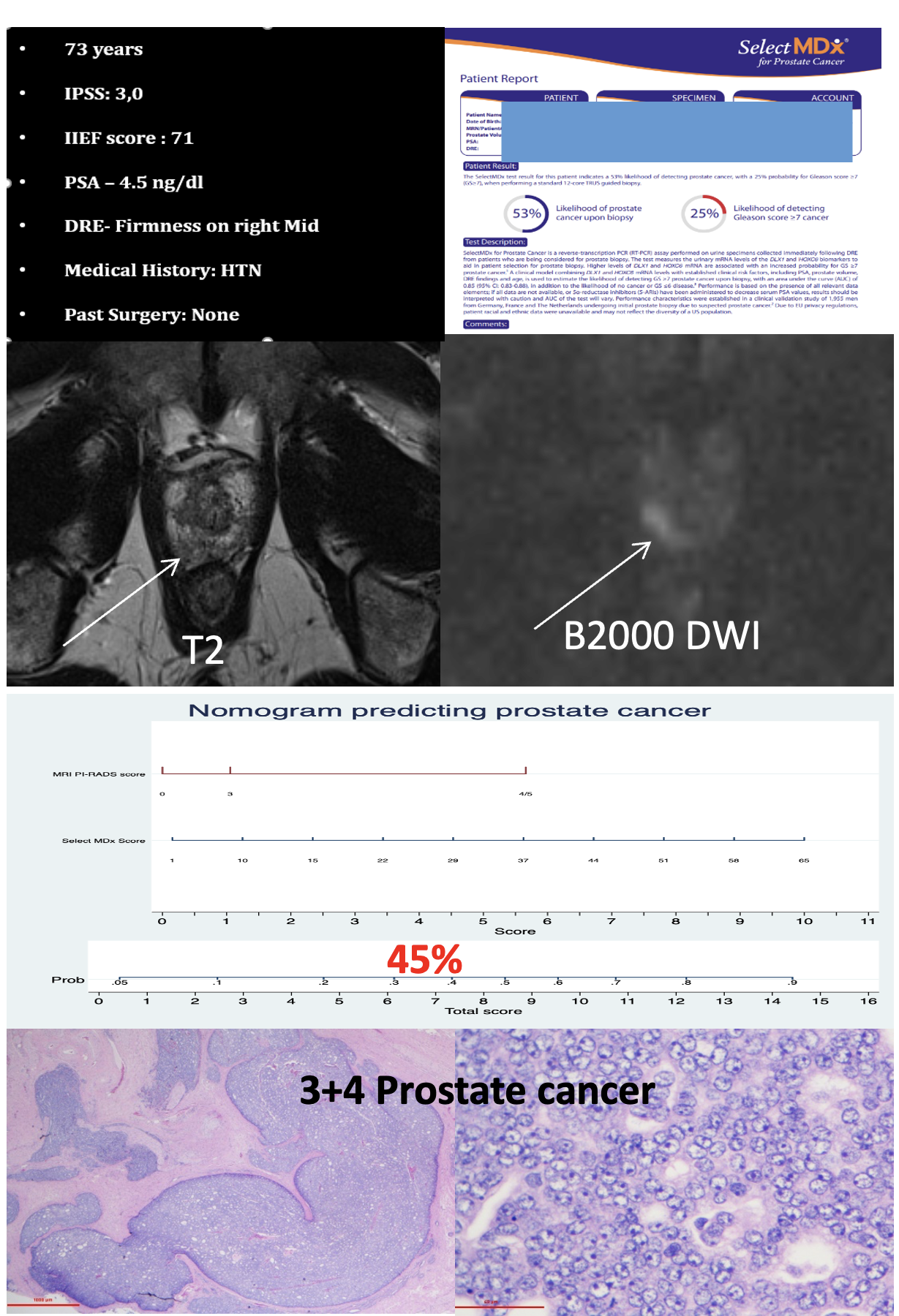

Supplement: Supplementary file 1 — Supplimentary Figure Case scenario showing patient information, Select MDx score information, MRI information and biopsy information. 73 years old gentleman with PSA 4.5ng/dL and elevated Select MDx score underwent MRI prostate that showed PI‐RADS 4 on right midgland of prostate. Nomogram showed 45% risk of prostate cancer.Patient underwent biopsy and found to have Gleason 3+4 prostate cancer. [file CNR2-6-e1668-s001.tiff]
